# White matter injury but not germinal matrix hemorrhage induces elevated osteopontin expression in human preterm brains

**DOI:** 10.1186/s40478-021-01267-7

**Published:** 2021-10-15

**Authors:** Gisela Nilsson, Ana A. Baburamani, Mary A. Rutherford, Changlian Zhu, Carina Mallard, Henrik Hagberg, Regina Vontell, Xiaoyang Wang

**Affiliations:** 1 Centre of Perinatal Medicine and Health, Institute of Neuroscience and Physiology, Department of Physiology, Sahlgrenska Academy, University of Gothenburg, 40530 Gothenburg, Sweden; 2 Centre for the Developing Brain, Department of Perinatal Imaging and Health, School of Biomedical Engineering and Imaging Sciences, King’s College London, King’s Health Partners, St Thomas’ Hospital, London, UK; 3 Department of Clinical Neuroscience, Center for Brain Repair and Rehabilitation, Institute of Neuroscience and Physiology, University of Gothenburg, Gothenburg, Sweden; 4 Henan Key Laboratory of Child Brain Injury and Pediatric Clinical Research Center, Institute of Neuroscience and Third Affiliated Hospital of Zhengzhou University, Zhengzhou, China; 5 Centre of Perinatal Medicine and Health, Institute of Clinical Sciences, Department of Obstetrics and Gynecology, Sahlgrenska Academy, Gothenburg University, 40530 Gothenburg, Sweden; 6 University of Miami Brain Endowment Bank, Miami, FL 33136 USA

**Keywords:** Osteopontin, Preterm, Postmortem brain, Microglia, White matter, Hemorrhage

## Abstract

Osteopontin (OPN) is a matricellular protein that mediates various physiological functions and is implicated in neuroinflammation, myelination, and perinatal brain injury. However, its expression in association with brain injury in preterm infants is unexplored. Here we examined the expression of OPN in postmortem brains of preterm infants and explored how this expression is affected in brain injury. We analyzed brain sections from cases with white matter injury (WMI) and cases with germinal matrix hemorrhage (GMH) and compared them to control cases having no brain injury. WMI cases displayed moderate to severe tissue injury in the periventricular and deep white matter that was accompanied by an increase of microglia with amoeboid morphology. Apart from visible hemorrhage in the germinal matrix, GMH cases displayed diffuse white matter injury in the periventricular and deep white matter. In non-injured preterm brains, OPN was expressed at low levels in microglia, astrocytes, and oligodendrocytes. OPN expression was significantly increased in regions with white matter injury in both WMI cases and GMH cases. The main cellular source of OPN in white matter injury areas was amoeboid microglia, although a significant increase was also observed in astrocytes in WMI cases. OPN was not expressed in the germinal matrix of any case, regardless of whether there was hemorrhage. In conclusion, preterm brain injury induces elevated OPN expression in microglia and astrocytes, and this increase is found in sites closely related to injury in the white matter regions but not with the hemorrhage site in the germinal matrix. Thus, it appears that OPN takes part in the inflammatory process in white matter injury in preterm infants, and these findings facilitate our understanding of OPN’s role under both physiological and pathological conditions in the human brain that may lead to greater elucidation of disease mechanisms and potentially better treatment strategies.

## Introduction

Preterm infants born before 32 weeks’ gestation or with a birth weight less than 1,500 g are frequently affected by brain injuries that are associated with long-term neurological complications such as cerebral palsy, vision loss, hearing problems, impaired learning, and behavioral and psychological problems [35, 61]. The major types of brain injury affecting preterm infants are white matter injury and germinal matrix-intraventricular hemorrhage (GMH-IVH) [9, 67].

Injury affecting the white matter adjacent to the cerebral ventricles, referred to as periventricular white matter injury (PVWMI), is the most common form of white matter injury in preterm infants and is caused by developmental immaturity, infection, and hypoxia/ischemia. These insults induce cell death, excitotoxicity, free-radical accumulation, energy failure, and inflammation that lead to brain lesions with focal necrosis involving reactive astrogliosis and microglia activation as well as to a decrease in pre-myelinating oligodendrocytes [72, 74]. PVWMI can be diffusely apparent or more severe, involving cyst formation. On diagnostic magnetic resonance imaging scans, WMI is visualized as either discrete focal or more diffuse areas of magnetic resonance signal abnormalities [19, 30, 42]. The white matter in premature infants is rich in oligodendrocyte progenitors and pre-myelinating oligodendrocytes that are vulnerable to excitotoxicity, oxidative stress, and inflammation, all of which can lead to oligodendrocyte developmental arrest and disrupted myelination and white matter injury [6, 24, 46].

GMH-IVH is a consequence of the fragile vascular network of the germinal matrix and immature cerebral autoregulation [8, 9]. GMH usually develops between the thalamus and the caudate, affecting the highly vascularized subependymal germinal matrix area, and if large it can rupture into the ventricles (IVH) [47]. GMH results in pressure on surrounding tissue, partial secondary ischemia, blood clot formation, and disruption of cerebrospinal fluid circulation, all of which can contribute to secondary injury. In addition, GMH-IVH can further trigger microglia activation, reactive astrogliosis, and inflammatory responses [7, 32, 38] in adjacent white matter also contributing to white matter injury [9, 37, 62].

Osteopontin (OPN) is a multifunctional extracellular matrix glycoprotein and cytokine that is involved in many physiological and pathological processes, including cell signaling, tissue repair, bone formation, immunity, and inflammation [13, 20]. The many functions of OPN may reflect its multiple variants arising from transcriptional, posttranscriptional, and posttranslational modifications as well as the diversity of cell types that OPN interacts with. Under physiological conditions, the expression of OPN in various organs is low but its expression can be upregulated in response to injury or inflammation [70]. In the central nervous system (CNS), OPN exerts neuroprotective functions [14, 45] and can have detrimental effects in neuroinflammation [14, 69]. OPN has been shown to act as a chemoattractant for microglia, macrophages, and astrocytes during glial scar formation after ischemic injury in adult rodent models [21] and to play a role in neurodegeneration and demyelination diseases such as multiple sclerosis [12, 15, 18, 44, 76] as well as in re-myelination [55]. Increased levels of OPN transcripts were reported in the brain tissue of multiple sclerosis patients [15]. In the immature brain, OPN is one of the most highly regulated molecules in perinatal brain injury [26, 68], where a neuroprotective role has been suggested in neonatal brain injury in rodent models equivalent to term infants [16, 68], while in younger mouse brains administration of OPN peptides exacerbated brain injury in a mouse model of hypoxia-ischemia-induced preterm brain injury [1]. In human infants, strong OPN immunoreactivity was detected in injured axons at the periphery of the ischemic zone in periventricular leukomalacia lesions in the subacute and chronic stages [65]. However, the expression and cellular source of OPN in human preterm infants, and its relation to brain injury, remains largely unknown. The aim of the present study was to explore the expression pattern of OPN in postmortem preterm infant brains and how it is affected in white matter injury and GMH.

## Material and methods

### Human postmortem brains

Written informed parental consent was acquired in accordance with the National Health Services (NHS) UK guidelines, and ethical approval was obtained from the National Research Ethics Services (West London), UK (ethic number: 07/H0707/139).

A total of 21 preterm postmortem brains at postmenstrual age 22–32 weeks from vaginally delivered infants were included in this study. Brain tissue blocks from these cases had a postmenstrual age calculated by gestational age (at delivery) plus age at death. No cases had known genetic mutations or diagnoses. Amniotic fluid infections were identified in 10/21 cases; however, no cases had identifiable vascular thrombosis or leptomeningitis. All brains were assessed macroscopically and microscopically at the time of clinical postmortem examination. Neuropathology and primary cause of death of the cases were evaluated by a perinatal pathologist. The causes of death were complications due to preterm birth/extreme prematurity such as pneumonia and congestive heart failure, and these were similar for all three groups of infants. The details of each case, including the neuropathological findings and clinical information, are summarized in Table 1.

**Table 1:** Summary of clinical information

Group and Case		Sex	GA at birth (weeks+ days)	Postnatal age	PMA at death (weeks+ days)	Birth weight (g)	Clinical context	Infection	Neuropathology
Control									
1		M	25 + 3	21 h 7 m	25 + 3	820	Ass IUGR (twin)	None	Edematous brain with transtentorial herniation of the unci, white matter patchy astrocytosis
2		M	23 + 6	9 h 51 m	23 + 6	520	Ass IUGR (twin)	None	Leptomeninges congested with focal hemorrhages, drop out of neurons
3		M	26 + 2	43 h	26 + 3	511.4	TTF (triplet), Ass IUGR, pulmonary hemorrhage	None	None
4		F	24 + 1	4 h 40 m	24 + 1	660	Oligohydramnios, extreme prematurity, congestive heart failure	AAAFI	None
5		M	23 + 4	IUD/Stillbirth	23 + 4	610		AAAFI	None
6		M	28 + 1	< 1 h	28 + 1	1308.4	Oligohydramnios, lung hypoplasia, congestive heart failure	None	None
7		M	22 + 0	TOP	22 + 0	401.2	Anhydramnios, renal agenesis	None	None
WMI									
8		M	24 + 0	5w 1d 8 h 25 m	29 + 1	1010	Retroplacental hemorrhage	AAAFI, necrotizing enterocolitis	PVMWI, patchy WM gliosis, oedema, laminar necrosis, and neuron loss (HI)
9		M	29 + 3	11 h 17 m	29 + 3	1630	TTF, hydrops fetalis	AAAFI	PVWMI, patchy reactive astrocytosis, drop out of neurons
10		M	26 + 5	1d 7 h 52 m	26 + 6	641.4	Ass IUGR (twin), oligohydramnios, congestive heart failure	None	PVWMI, cerebellar hemorrhages, oedema, white matter gliosis
11		M	24 + 6	16d 19 h 10 m	27 + 0	715.4	PPROM, acute necrotizing pneumonia	AAAFI	PVWMI, focal neuron loss (HI), drop out of neurons, oedema
12		M	26 + 6	2d 20 h 46 m	27 + 1	807.6	TTF	None	Oedema, degenerating neurons, perivascular hemorrhages, neuron loss, apoptotic neurons, PVL (parietal)
13		F	27 + 5	22d	30 + 6	1440	Placental hemorrhage, congestive heart failure with edematous	None	Extensive PVL (on MRI), mildly dilated ventricles, drop out of neurons, oedema
14		F	27 + 0	7d	28 + 0	858.6	IUGR, congestive heart failure	Necrotizing enterocolitis	Oedema, focal neuronal loss (HI), patchy gliosis in PVWM, PVL (on MRI) right IVH (grade II) – on cranial US
GMH	Grade								
15	II	M	22 + 0	NND	22 + 0	476	Congestive heart failure	AAAFI/ Chorio-amnionitis	IVH/GMH, oedema, astrocytosis in white matter of dentate, focal neuronal loss in BG
16	I	M	27 + 5	10d 11 h 28 m	29 + 1	940	Congestive heart failure	AAAFI	Bilateral GMH/IVH (in situ)
17	III	M	24 + 0	4w 6d 19 h 12 m	28 + 6	596	Acute pneumonia, heart failure	AAAFI	Multiple GMH with focal rupture, oedema, patchy astrocytosis, some neuronal loss
18	I	F	26 + 2	18d	28 + 6	1126.8	Ass IUGR, congestive heart failure	Necrotizing enterocolitis	Focal GMH, hemorrhage of right cerebellum WM gliosis
19	III	M	24 + 6	53d	32 + 3	1637	Congestive heart failure	CMV (mild)	GMH, patchy WM gliosis, focal neuronal loss (HI), mild ventriculomegaly
20	II	M	22 + 5	4 h 9 m	22 + 5	530	Mild retroplacental hemorrhage	AAAFI	GMH, oedema
21	IV	F	24 + 1	2d 3 h 37 m	24 + 3	460	Prematurity, congestive heart failure, Ass IUGR	None	GMH, oedema

Gestational age (GA), postmenstrual age (PMA), asymmetric intrauterine growth restriction (Ass IUGR), acute ascending amniotic fluid infection (AAAFI), white matter injury (WMI), germinal matrix hemorrhage (GMH), hypoxic-ischemic (HI), intraventricular hemorrhage (IVH), intrauterine death (IUD), periventricular white matter injury (PVWMI), periventricular leukomalacia (PVL), preterm premature rupture of the membranes (PPROM), termination of pregnancy (TOP), Twin-Twin Transfusion (TTF), neonatal death (NND). *Drop out of neurons refers to Ammons’s horn, Purkijne cells, and dentate. GMH grades: I: hemorrhage confined to the GM, II: intraventricular extension involving ˂50% of the ventricle, III: extension of hemorrhage into a dilated ventricle or intraventricular extension involving ˃50% of the ventricle, IV: extension of hemorrhage into the surrounding parenchyma

### Tissue preparation

Following postmortem examinations, whole brains were fixed with 4% formalin for 5–7 weeks depending on their size. The whole brains were sliced by a pathologist, and tissue blocks were processed on a Bright Tissue Processor (Bright Instrument Co. Ltd.). Paraffin-embedded tissue blocks of the frontal lobe at the level of the caudate (i.e., anterior to Ammon’s horn) were sectioned coronally at 6 µm using a Leica RM2245 microtome (Leica Microsystems Ltd.) on super frost plus slides (Thermo Fisher, UK) [62, 73]. One section per case was used for routine hematoxylin and eosin (H&E) staining to assess the gross neuropathology. Adjacent sections were then used for immunohistochemical and immunofluorescent staining.

### Immunohistochemistry and immunofluorescent staining

As previously described [73] human postmortem sections underwent routine paraffin removal and rehydration, were immersed in preheated 10 mM citric acid (pH 6.0) with 0.1% Tween-20 (VWR International Ltd.) for 30 min, and were cooled to room temperature for 20 min. Slides were washed with phosphate-buffered saline (PBS; pH 7.4) and placed in 3% hydrogen peroxide to quench endogenous peroxidase activity. Sections were blocked with 5% fetal bovine serum, and primary antibodies were incubated overnight at 4 °C. Details of the primary antibodies are given in Table 2.

**Table 2:** Primary and secondary antibody information

Antigen	Catalogue Number	Source	Species	Dilution [ab conc.]	Target
Osteopontin (IgG2a)	ab69498	Abcam	Mouse	1:750	Osteopontin
GFAP (IgG1)	G3893	Sigma	Mouse	1:1000	Astrocytes, radial glia
Olig2	AB9610	Millipore	Rabbit	1:500	Oligodendrocytes
Iba1	019–19,741	WAKO	Rabbit	1:1000	Microglia
Biotinylated secondary	BA-2001	Vector Laboratories	Horse anti-mouse	1:200 [1.5 mg/ml]	
ABC Elite Kit	PK6200	Vector Laboratories		1:200	
Alexa Fluor 488	A21131	Life Technologies	Goat anti-mouse (IgG2a)	1:500 [2 mg/ml]	
Alexa Fluor 546	A21123	Life Technologies	Goat anti-mouse (IgG1)	1:500 [2 mg/ml]	
Alexa Fluor 546	A11010	Life Technologies	Goat anti-rabbit (IgG)	1:500 [2 mg/ml]	

For immunohistochemical staining of OPN on the following day, sections were incubated with biotinylated horse anti-mouse secondary antibodies (1:200, Vector Laboratories) for 1 h at room temperature, washed with PBS, and then incubated for 1 h with avidin–biotin complex (ABC, 1:200, Vector Laboratories, UK). The reactions were visualized with 3,3’-diamino-benzidine (DAB; Thermo Fisher Scientific, UK) for 10 min. Sections were then dehydrated, cleared in xylene, and cover slipped.

For calculation of the number of OPN-positive cells, we used a light microscope (Olympus, BX56) modified for stereology with a motorized stage (H1P4BX ProScan stage with V31XYZE ProScan III Controller) with newCAST software (Visiopharm, Hørsholm, Denmark). Delineation of the region of interest (ROI) was performed using a 1.25 × objective lens. Frames were randomly arranged within the ROI by the software using a 20 × objective lens covering 15% of the ROI, and the images of the frames were used to calculate the number of cells using Fiji ImageJ.

For immunofluorescent staining (OPN-Iba1, OPN-GFAP, and OPN-Olig2), sections were prepared as described above, and the next day the sections were rinsed in PBS and then incubated in a secondary antibody cocktail (Table 2) for 2 h at room temperature. Sections were washed and then mounted using ProLong Gold with DAPI (Invitrogen) and cover slipped.

For cell density calculations, four images were acquired within the white matter injury region using a 20 × objective lens on a Zeiss Axio Imager Z2 microscope, and the number of cells was counted using Fiji ImageJ.

### Statistics

GraphPad Prism version 8.3.0 (GraphPad software, San Diego, CA, USA) was used to perform the statistical analyses. One-way ANOVA was used for all comparisons. The data are presented in bar graphs as the mean ± SEM. *P* < 0.05 was considered statistically significant.

## Results

### Characteristics of white matter injury

**Fig. 1:**
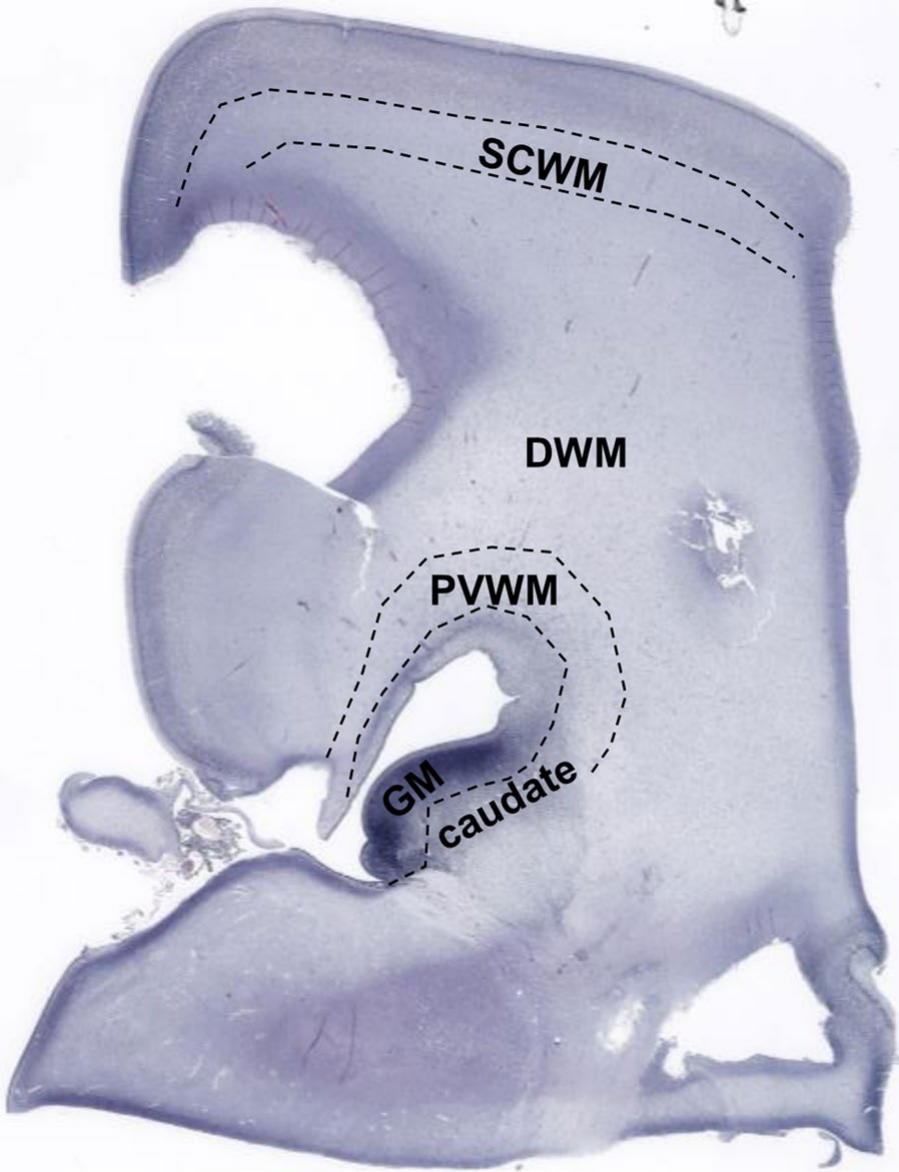
Global view of an H&E-stained coronal section anterior to Ammon´s horn at 26 + ^6/7^ weeks’ postmenstrual age. SCWM; sub-cortical white matter, DMW; deep white matter, PVMI; periventricular white matter, GM; germinal matrix

**Fig. 2:**
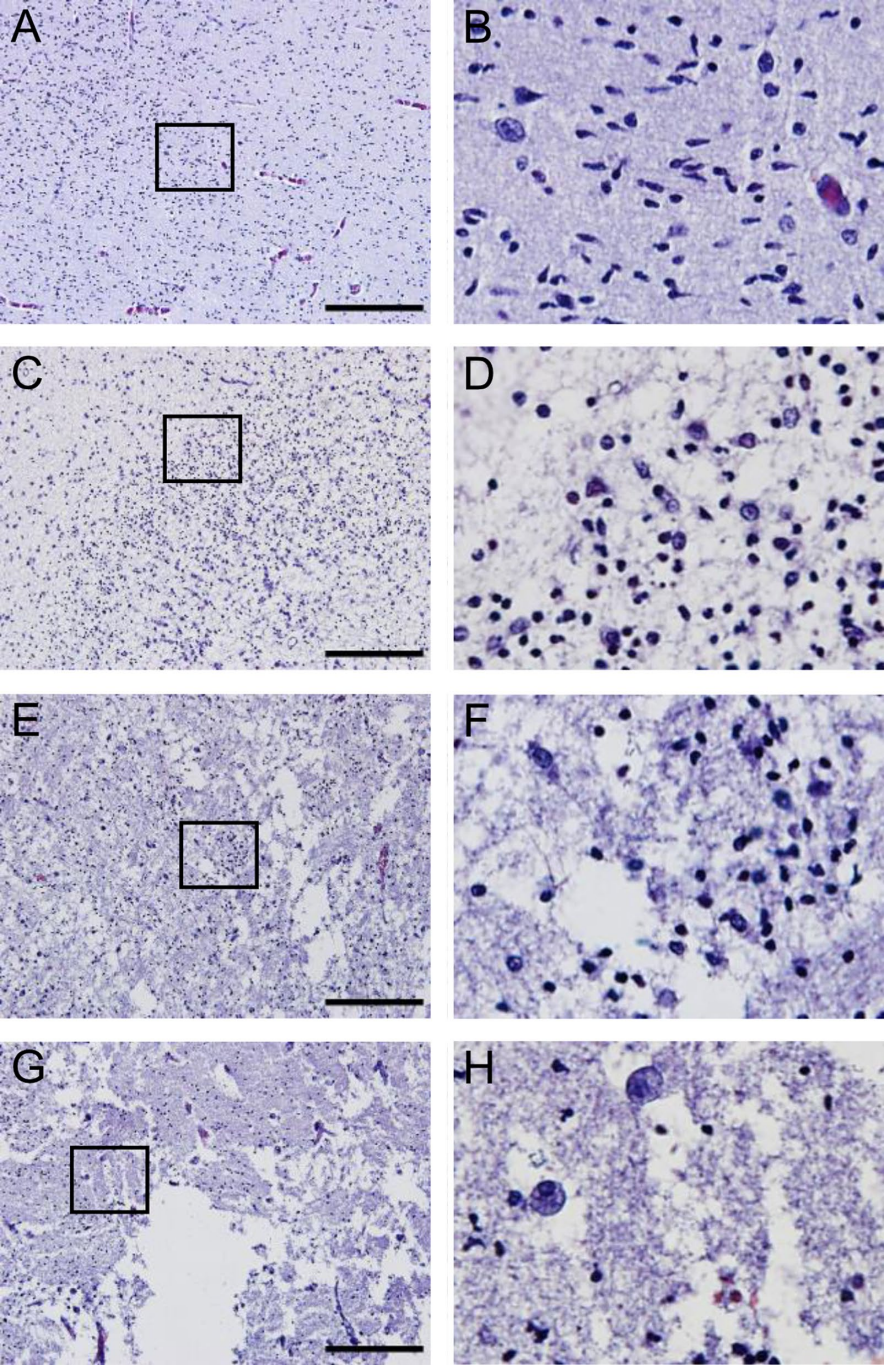
The degree of WMI assessed in H&E-stained sections. Representative images of white matter with no detectable injury (**A–B**), diffuse injury displaying disrupted tissue structures, hypercellularity, and pyknotic cells (**C–D**), moderate injury with patchy tissue loss and patches of pyknotic cells (**E**–**F**), or severe injury with extensive tissue loss (**G–H**). Right panel: magnification of the selected area. Scale bar = 200 µm

Three groups of preterm infants at postmenstrual age 22–32 weeks were included in this study, including 7 control cases, 7 WMI cases, and 7 GMH cases (Table 1). Brain sections at the level of the caudate (i.e., anterior to Ammon’s horn) were stained with H&E to assess the gross neuropathologies. Figure 1 shows an overview of a brain section where injury was assessed in different regions of the white matter; the periventricular white matter, the deep white matter, and the sub-cortical white matter. White matter regions with intact tissue structures were considered as having no WMI (Fig. 2A–B). Pyknotic cells were present in non-injured white matter as expected because apoptosis plays a role in normal development by regulating cell numbers [36]. However, white matter regions with diffuse injury, i.e., disrupted tissue structures, were accompanied by an increase of pyknotic cells and hypercellularity (Fig. 2C–D). White matter regions with moderate injury showed patchy tissue loss and patches of pyknotic cells (Fig. 2E–F). In white matter regions with severe injury, extensive tissue loss was observed (Fig. 2G–H). In the WMI cases, the tissue damage was more pronounced in the periventricular white matter compared to the deep white matter. In the cases with moderate to severe periventricular WMI, the deep white matter also showed diffuse to moderate injury, sometimes extending to the sub-cortical white matter. In addition, GMH was accompanied by WMI in all GMH cases used in the current study, although to a lesser extent compared to WMI cases. WMI in GMH cases was also predominately located in the periventricular white matter, extending to the deep white matter and less so to the sub-cortical white matter.

### Characteristics of GMH

**Fig. 3:**
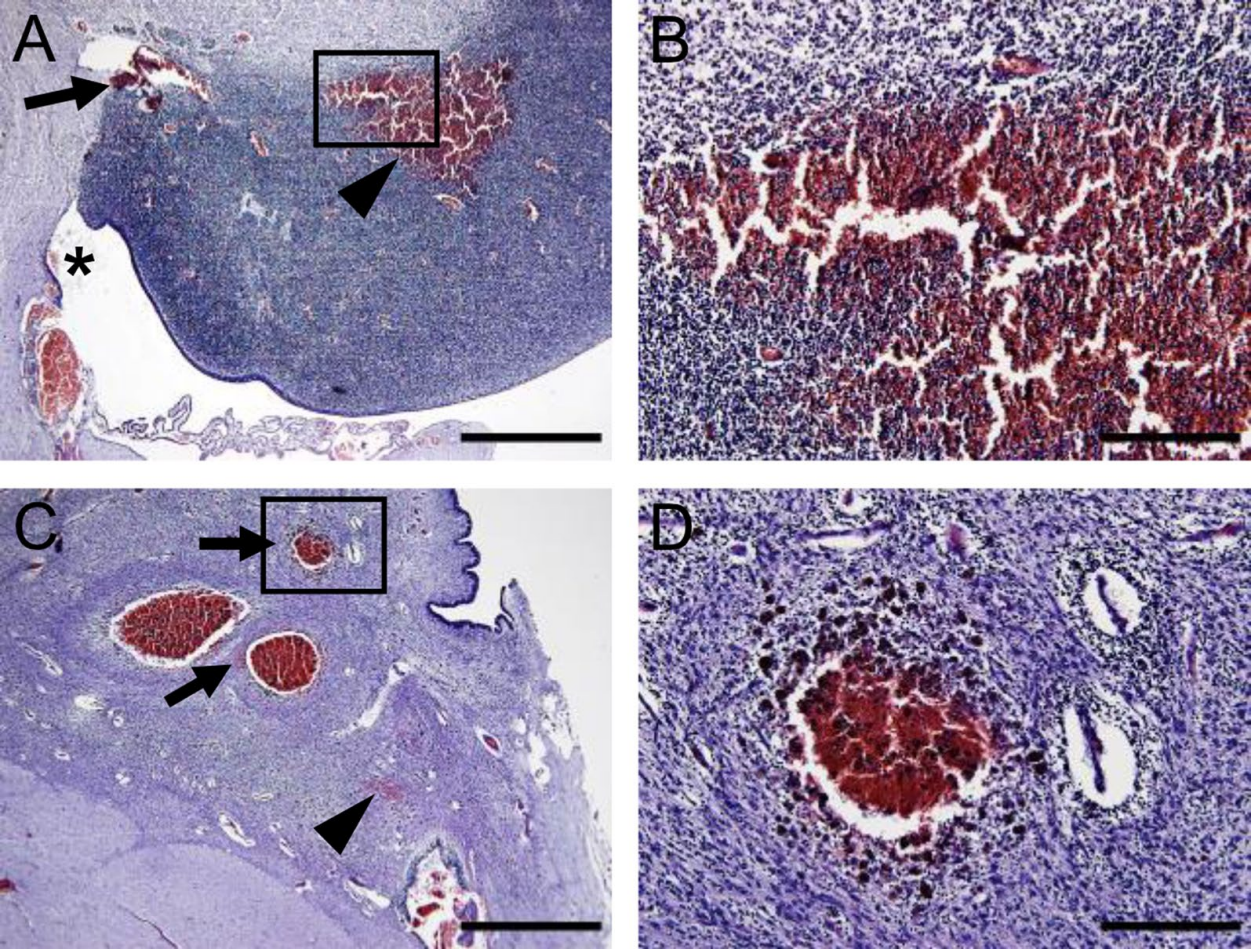
Hemorrhage in the germinal matrix as observed in H&E-stained sections. **A–B** GMH in a case at postmenstrual age 22 + ^5/7^ weeks detected as dispersed (arrowhead) or limited around vessels (arrow) as well as apparent in the ventricle (star). **C–D** In a GMH case at postmenstrual age 32 + ^3/7^ weeks the hemorrhage was mainly detected around ruptured vessels and contained aggregates (arrow), but small, dispersed bleedings (arrowhead) were also noticeable. Scale bars: (**A** and** C**) = 1 mm, (**B** and **D**) = 200 µm

Grade of hemorrhage in the GMH cases is shown in Table 1. We could observe the hemorrhage in the brain sections of 5 out of the 7 GMH cases used in this study. In one of the cases without observed hemorrhage, only a small part of the germinal matrix was present in the section, while in the other case hemorrhage was observed in the periventricular white matter, but these cases were included in this group because hemorrhages were observable at post-mortem in brain sections not used in the current study. The extent of the hemorrhage observed in the sections ranged from small to large (Fig. 3). In 3 out of the 7 GMH cases, IVH was observed in the sections. In the germinal matrix, the hemorrhage was visible as dispersed (Fig. 3A–B) or as limited around ruptured vessels (Fig. 3C–D).

### Amoeboid microglia are increased in WMI

The typical morphology of microglia in resting condition is ramified with many fine short processes for sensing their surroundings. During development and in response to injury, they take on an amoeboid morphology with rounded cell bodies and little or no processes, and this leads to increased phagocytic capability and increased motility [11]. We examined the cell density and morphology of microglia using the microglia marker ionized calcium-binding adapter molecule 1 (Iba1), a marker for ramified, intermediate, and amoeboid/activated microglia [33, 62, 66, 71].

**Fig. 4:**
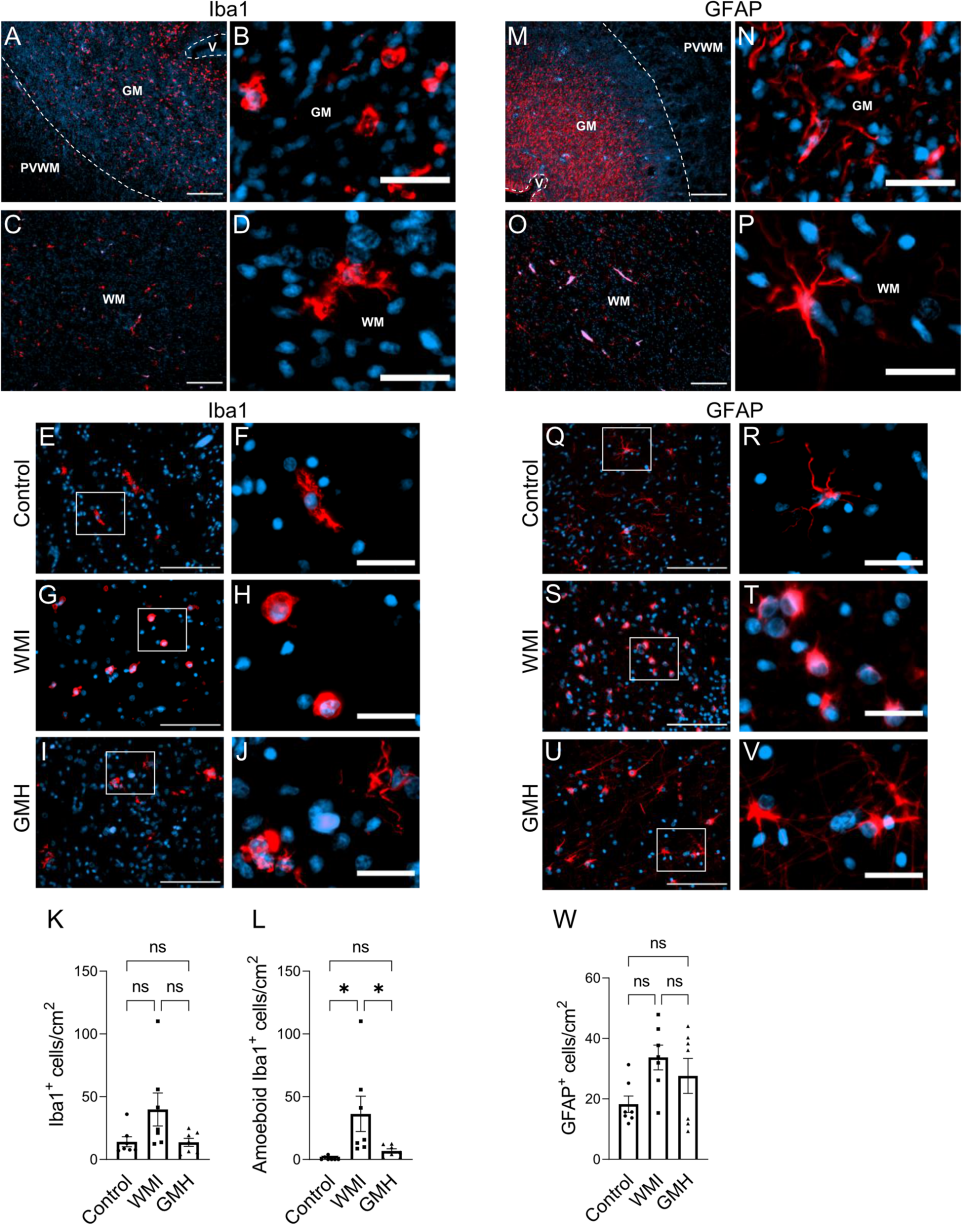
Microglia and astrocytes are altered in WMI. (**A**-**J**) Iba1 immunostaining (red) and nuclear counterstaining with DAPI was performed. (**A–C**) Examples demonstrating the distribution and morphology of microglia in the germinal matrix (**A**-**B**) and in the white matter (**C**-**D**) in non-injured tissue. (**E**-**J**) Representative immunofluorescence images demonstrating microglia in white matter having ramified morphology in control, amoeboid morphology in WMI, and ramified to intermediate morphology in GMH. (**K**) The numbers of Iba1^+^ cells in the white matter. (**L**) The numbers of amoeboid Iba1^+^ cells in the white matter. (**M**-**V**) Immunostaining with the astrocyte marker GFAP (red) and nuclear counterstaining with DAPI. (**M–P**) Images showing the distribution and morphology of astrocytes in non-injured tissue in the germinal matrix (**M–N**) and white matter (**O–P**). (**Q–V**) Representative images showing GFAP^+^ astrocytes in the white matter having stellate morphology in controls, while having different degrees of reactive morphology in WMI and GMH. (**W**) The numbers of GFAP^+^ cells in the white matter. Scale bars: (**A** and** C**) = 200 µm, (**B **and** D**) = 25 µm, (**E**,** G**, and** I**) = 50 µm, (**F**, ** H**, and** J**) = 25 µm, (**M **and** O**) = 200 µm, (**N **and** P**) = 25 µm, (**Q**, **S**, and **U**) = 50 µm, (**R**, **T**, and** V**) = 25 µm

In control cases, microglia were numerous in the germinal matrix and exhibited an amoeboid morphology (Fig. 4A–B), and this was also true for the WMI and GMH cases. In non-injured white matter, the microglia displayed a ramified morphology with extensive complex branching (Fig. 4C–F), while the microglia showed an amoeboid morphology with round to amorphous structures with a variety of short pseudopodia in WMI cases (Fig. 4G–H) and an intermediate or ramified morphology in the GMH cases (Fig. 4I–J). Comparing the numbers of microglia in the white matter injury regions, we did not find a significant difference in the numbers of Iba1-positive cells between the three groups (Fig. 4K). Nevertheless, an increase in microglia numbers was found in 5 out of the 7 WMI cases. However, there was a significant increase in the number of Iba1-positive cells having an amoeboid morphology in the WMI cases compared to the control cases that was not observed in the GMH cases (Fig. 4L), suggesting that the microglial morphological state is related to the severity of the WMI.

### Astrocytes are altered in WMI

Astrocytes react to WMI and undergo morphological, molecular, and functional changes through reactive astrogliosis [5, 59]. For examination of the numbers and morphology of astrocytes, we used the astrocyte marker glial fibrillary acidic protein (GFAP). In non-injured tissue, GFAP-positive astrocytes were most abundant in the germinal matrix, exhibiting a fibrous and superficial morphology (Fig. 4M–N), and they were observed in periventricular crossroads and around blood vessels. GFAP-positive astrocytes were less abundant in white matter where they had a stellate morphology (Fig. 4O–P). In WMI and GMH cases, GFAP-positive astrocytes in the white matter displayed a morphology indicating varying degrees of reactivity, with elaborated processes and extended cell bodies (Fig. 4S–V). This was most obvious in regions close to the injury. However, there was no significant difference in overall cell numbers between the groups (Fig. 4W), although cases with moderate to severe WMI showed increased astrocyte numbers (data not shown).

### Increased OPN expression is found in WMI regions

**Fig. 5:**
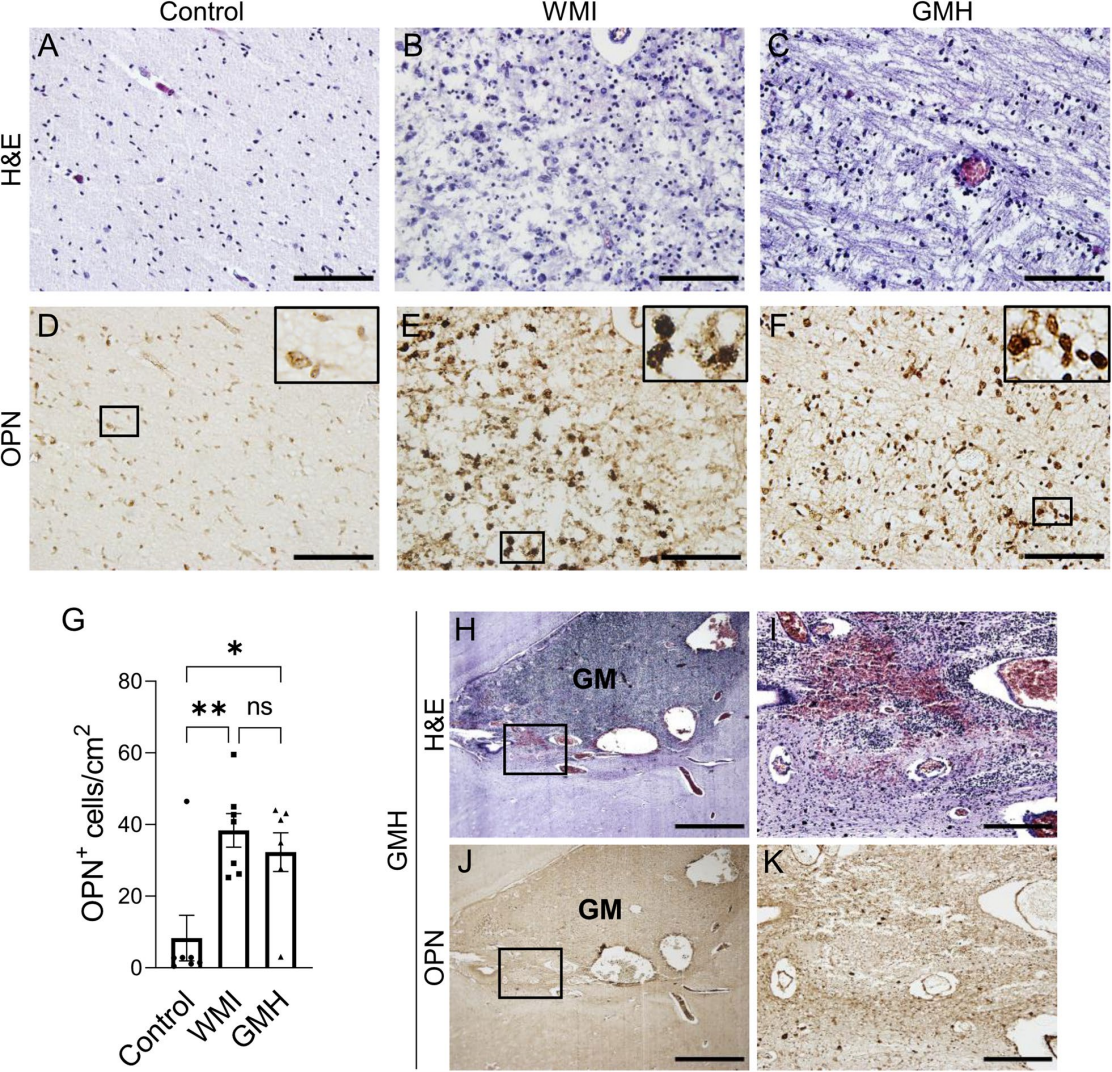
OPN expression in the white matter and the germinal matrix. Parallel sections from a representative control, WMI, and GMH case stained with H&E (**A**-**C**) or OPN-DAB (**D**-**F**) demonstrating tissue structure and OPN^+^ cells in regions with white matter injury. (**G**) The numbers of OPN^+^ cells in the white matter. (**H** and** J**) Parallel sections from a GMH case at postmenstrual age 28 + ^6/7^ weeks stained with H&E or OPN-DAB. Scale bars: (**A**-**F**) = 200 µm, (**H** and** J**) = 1 mm, and (**I** and** K**) = 200 µm

To investigate the expression of OPN in the developing brain and in response to brain injury, parallel sections to the H&E-stained sections were stained for OPN. Overall, the expression of OPN in the control cases was low in white matter regions, including the periventricular white matter, deep white matter, and sub-cortical white matter. In WMI cases, OPN-positive cells were found mainly in the white matter in association with injury, with more staining observed in regions with moderate to severe injury compared to diffusely injured white matter. OPN-positive cells were also found in regions with WMI in all but one of the GMH cases (Fig. 5A-F). There was a significant increase in OPN-positive cells in the regions of WMI in both the WMI (p = 0.003) and GMH cases (p = 0.017) compared to the corresponding white matter regions in control cases (Fig. 5G). This suggests that OPN expression in the white matter in the non-injured developing brain is low and that it is increased following WMI in preterm infants.

### OPN expression is not elevated in regions of germinal matrix hemorrhage

OPN expression has been shown to be associated with different kinds of injury, and we next investigated if OPN expression is also elevated in association with hemorrhage regions as was found in WMI. OPN staining was negative in the germinal matrix in all control and WMI cases. Only very few OPN-positive cells were found in the germinal matrix following hemorrhage in the GMH group (Fig. 5H–K), suggesting that OPN expression is not induced in association with the hemorrhage sites.

### Elevated OPN expression is induced in microglia and astrocytes, but not in oligodendrocytes

To identify the cellular source of OPN in the preterm infant brains, we first examined the expression of OPN in the control cases using double fluorescent immunostaining of OPN together with Iba1 (microglia), GFAP (astrocytes), and the oligodendrocyte-specific marker Olig2, respectively. We found that OPN in white matter was co-expressed with all three cell types examined, namely microglia, astrocytes, and oligodendrocytes, although the expression level was low.

**Fig. 6:**
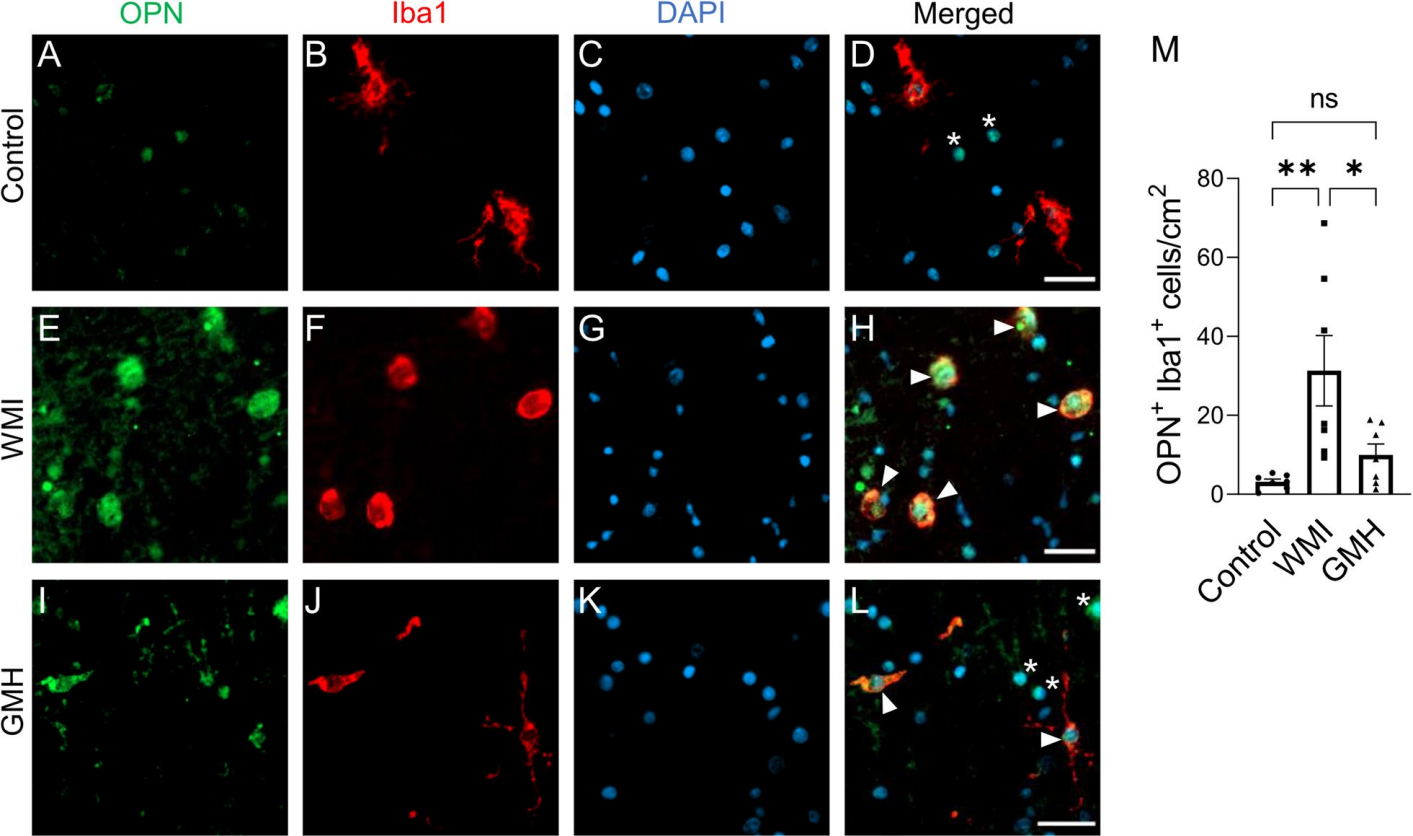
OPN expression is induced in amoeboid microglia. Double labeling of anti-OPN (green) and anti-Iba1 (red) with DAPI nuclear counterstaining was performed, and double-positive cells were counted in the white matter. (**A**-**L**) Representative immunofluorescence images from control (**A**-**D**), WMI (**E**–**H**), and GMH (**I**-**L**) cases (arrowhead: OPN-Iba1 double-labeled cells, star: OPN single-labeled cells). (**M**) Density of OPN^+^-Iba1^+^ cells. Scale bar = 25 µm

**Fig. 7:**
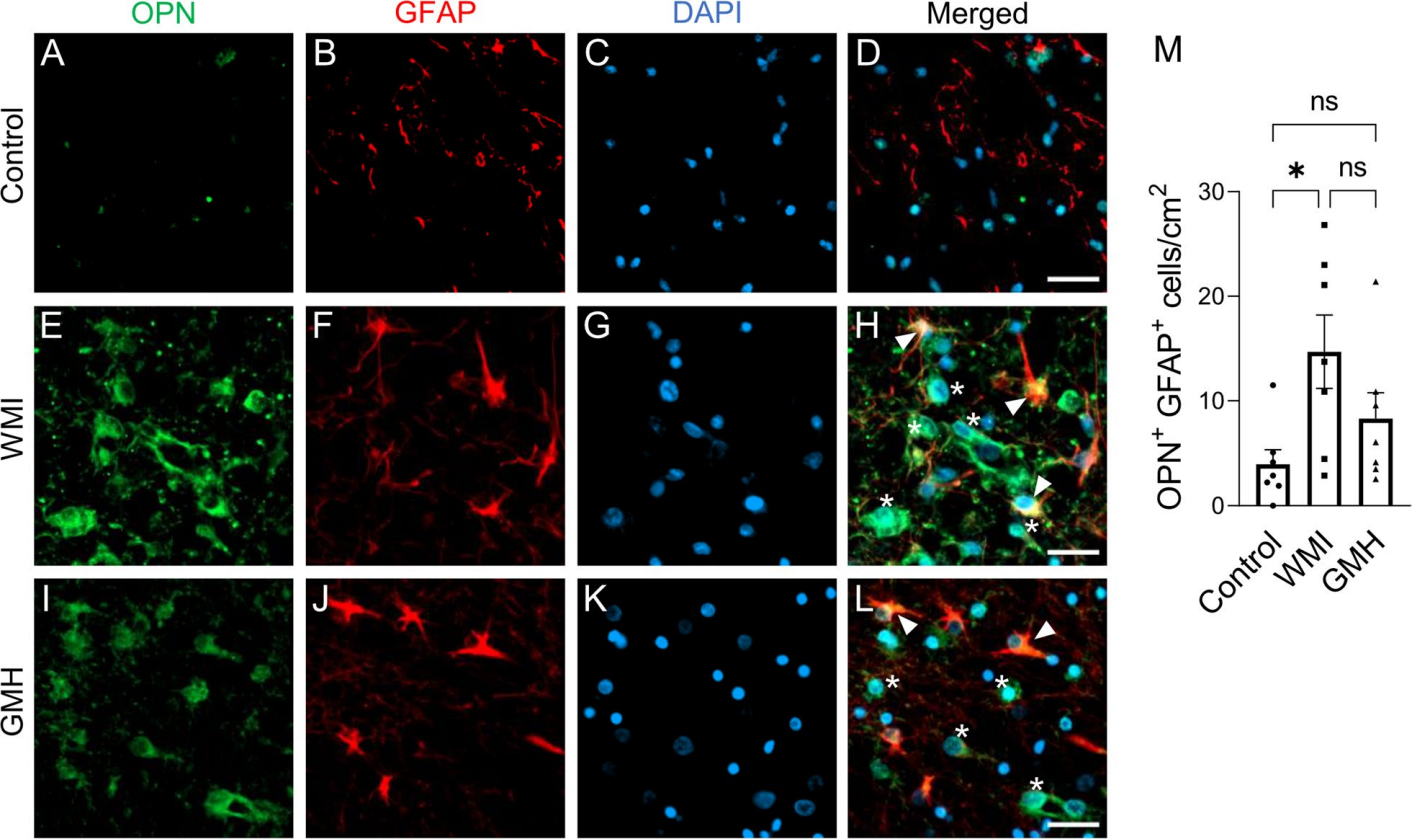
Astrocytes in WMI have increased expression of OPN. Staining of anti-OPN (green) and anti-GFAP (red) with DAPI nuclear counterstaining was performed and OPN-expressing astrocytes in the white matter were counted. (**A**-**L**) Examples of OPN-GFAP double-labeled cells in control (**A–D**), WMI (**E**–**H**), and GMH (**I**-**L**) cases (arrowhead: OPN-GFAP double-labeled cells, star: OPN single-labeled cells). (**M**) Density of OPN^+^-GFAP^+^ cells. Scale bar = 25 µm

**Fig. 8:**
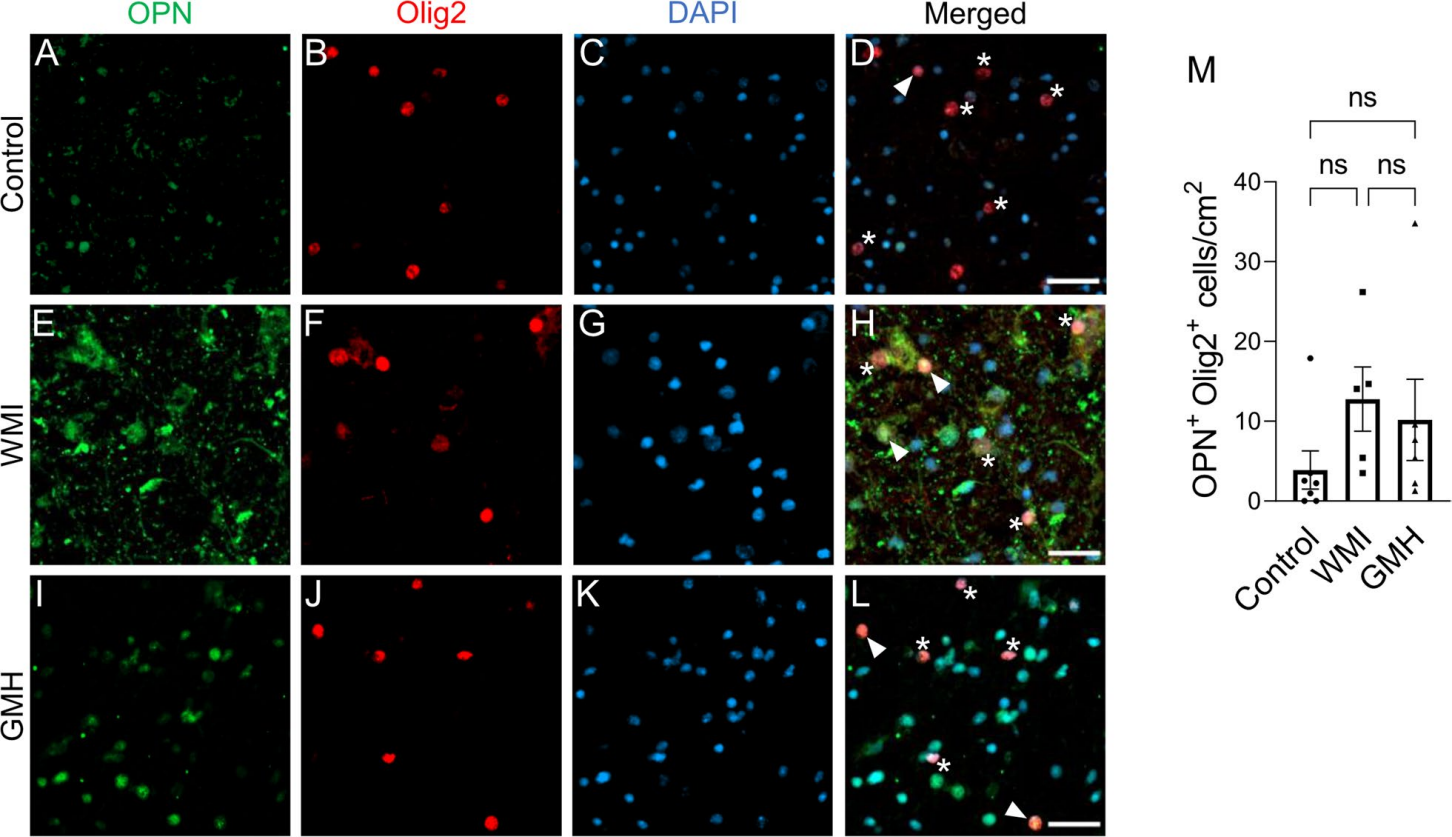
OPN expression in oligodendrocytes is not increased in response to white matter injury. Oligodendrocytes were stained for anti-Olig2 (red) and anti-OPN (green) along with DAPI nuclear counterstaining, and the number of double-labeled cells in the white matter was counted. (**A**-**L**) Representative immunofluorescence images from control (**A–D**), WMI (**E**–**H**), and GMH (**I**-**L**) cases (arrowhead: OPN-Olig2 double-labeled cells, star: OPN single-labeled cells). (**M**) Density of OPN^+^-Olig2^+^ cells. Scale bar = 25 µm

OPN was found to be secreted by macrophages and microglia to modulate the inflammatory responses to different brain injuries [48, 53, 57, 64], and upregulation of OPN in reactive astrocytes has also been described [28, 29]. We found that in regions with injured white matter there was a significant increase in the numbers of microglia expressing OPN in the WMI cases compared to the control cases (*p* = 0.005) (Fig. 6A–H and M), while there was no significant increase in the numbers of OPN-expressing microglia associated with WMI regions in the GMH group compared to controls. However, microglia exhibiting ramified to amoeboid morphology in GMH cases expressed OPN (Fig. 6I–L and M). Similarly, in the white matter regions there was a significant increase in the numbers of astrocytes expressing OPN in WMI cases (*p* = 0.024), but not in GMH cases, compared to the control group (Fig. 7A–M). Furthermore, Olig2-positive oligodendrocytes co-expressed OPN but the number of OPN-positive oligodendrocytes in injured white matter did not change in WMI or GMH cases (Fig. 8A–M). This confirms that all three types of cells express OPN; however, OPN expression is increased in microglia and astrocytes but not in oligodendrocytes in response to WMI.

## Discussion

**Fig. 9:**
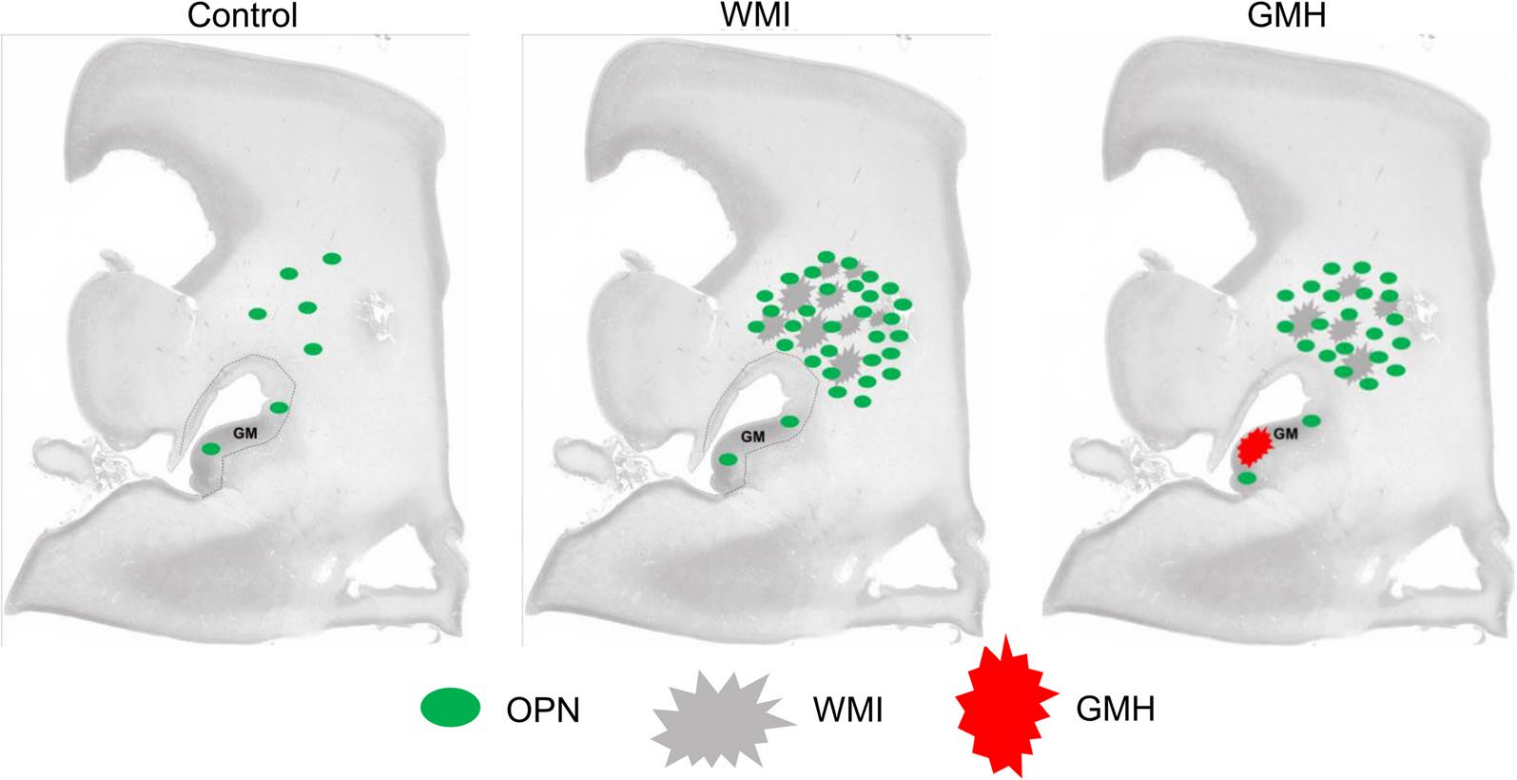
Summary figure illustrating the major findings. In the non-injured developing brain, the expression of OPN is low. In preterm brain injury, the expression of OPN is elevated, and this elevation is found in the injured white matter regions but not at the site of hemorrhage in the germinal matrix regions

In this study we show for the first time that the brain injury-induced increase in OPN expression in preterm infants is restricted to regions of WMI and is predominately seen in amoeboid microglia and astrocytes. In contrast, elevated OPN expression was not associated with hemorrhagic regions (Fig. 9). In the non-injured preterm brain, we found low expression of OPN in microglia, astrocytes, and oligodendrocytes. The expression of OPN in oligodendrocytes was unchanged in WMI.

In early brain development (23–35 weeks) microglia exhibit amoeboid features, while over time they acquire an intermediate morphology with few processes and ultimately mature into ramified microglia with small cell bodies and long processes [10]. The germinal matrix contains neuronal and glial precursors and is present in the foetal brain between 8 and 36 weeks’ gestation. Indeed, we observed that microglia in the germinal matrix regions displayed an amoeboid morphology irrespective of injury. Moreover, the germinal matrix was rich in microglia, as previously observed [62], and in astrocytes.

Microglia are implicated in perinatal brain injury, and one of the first cellular events in WMI in preterm infants is microglial transformation to an active amoeboid phenotype [43, 63, 71]. Via their activation, migration, phagocytosis, and expression of nitric oxide synthase, nitric oxide, and proinflammatory cytokines, they are suggested to be a contributing factor in mediating preterm brain injury [4, 24, 25, 62, 71]. As expected, we found a significant increase in amoeboid, supposedly activated, microglia in the injured white matter in WMI cases.

Astrocytes are associated with numerous functions during brain development, including blood brain barrier formation, synaptogenesis, neurotransmission, and metabolic regulation [60]. Mature astrocytes have many branches and fine processes, and they respond to multiple forms of brain insult, including perinatal brain injury, by changing their phenotype and morphology via reactive astrogliosis [52, 59]. Reactive astrogliosis is one of the hallmarks of WMI [27, 50] and is a reaction to inflammation and injury that can have both beneficial and detrimental consequences, leading to hypertrophy and in extreme cases leading to inflammation and to proliferation and scar formation at the borders of the tissue damage [59]. Like microglia activation, we found manifestations of reactive astrogliosis in proximity to WMI that appeared to be linked to the severity of injury, and preterm infants with severe injury displayed an increase in astrocytes exhibiting a morphology indicating reactivity.

GMH/IVH causes blood-induced pathological reactions such that the deposition of extravasated blood in the intraventricular space, which is followed by lysis of the red blood cells resulting in a subsequent release of extracellular hemoglobin throughout the brain, including in the periventricular white matter [39]. The accumulation of blood in the cerebral ventricles of infants with IVH also damages the adjacent white matter. All together, these events induce a series of subsequent pathological processes such as oxidative stress, inflammation, pressure-induced partial ischemia, perturbed signaling pathways, and remodeling of the extracellular matrix that lead to periventricular white matter damage [9]. Indeed, autopsy studies of brain samples have revealed evidence of white matter injury in 50–78% of premature infants with IVH [2, 51, 58]. The GMH cases in the current study had various grades of hemorrhage and displayed various degrees of WMI, although the injury was generally less severe compared to WMI cases. Six out of the seven GMH cases had an infection. Moreover, GMH itself causes inflammation [3, 9, 34] together with additional complications, e.g., infection, and these are among the known factors contributing to secondary white matter brain injury in premature infants [9], which might have contributed to the upregulation of OPN expression in the white matter areas that was observed in GMH cases. Inflammation and infection are known factors that cause increased OPN expression [20, 41, 54]. Increased OPN expression in turn regulates innate immune cells (macrophages and dendritic cells) and adaptive immune cells (T cells) at multiple levels (summarized and reviewed in [41]) and therefore plays an important role in the inflammatory response in multiple disease pathologies and injury processes, likely including preterm white matter injury.

Elevated OPN expression was observed in both WMI and GMH cases, and OPN immunoreactivity was restricted to the injured white matter regions. There was a significant increase in microglia expressing OPN in WMI cases, while this increase was not significant in GMH cases. This suggests that OPN is expressed predominately by microglia exhibiting an amoeboid, presumably activated phenotype, as these were more numerous in the WMI cases compared to the GMH cases. Indeed, we have previously shown that Iba1–positive microglia with amoeboid morphology are co-localized with CD68, a marker for microglia activation [33], in the periventricular white matter areas in preterm infants with GMH/IVH [62]. The number of astrocytes expressing OPN was not significantly increased in GMH cases as it was for WMI cases, and this may reflect that there were fewer reactive astrocytes in GMH cases that also displayed less injury in the white matter compared to the WMI cases. The potential correlations between WMI severity, GMH grade, or other complications and OPN expression could not be established because of the small number of cases used in this study.

OPN expression was not elevated in hemorrhage regions, and this may indicate that only certain types of inflammation, and not extravasated blood, lead to elevated OPN expression in the brain. In addition, we did not observe OPN-positive staining in the germinal matrix regions, even though microglia in the germinal matrix displayed amoeboid morphology regardless of injury. This is different from the microglia in regions adjacent to injury in the white matter, where the majority of the OPN staining was found. Such findings suggest that elevated OPN expression is linked to WMI, and microglia activation triggered by injury type-related features, such as inflammation, rather than the phenotypic state the cells are in during specific developmental processes. Together with the finding that OPN expression in oligodendrocytes did not change after injury, this may indicate that the elevated level of OPN expression in the human preterm infant brain may play a role in inflammatory processes after WMI. Indeed, in animal models of stroke and in in vitro models, induced OPN expression in activated microglia has been demonstrated to promote phagocytosis [53, 57, 64] and to modulate cytokine expression [48]. OPN has been reported to regulate the activation and function of microglia [75], while microglia have been shown to not only be involved in inflammation, but also to be important in oligodendrocyte and white matter development as well as in regeneration of myelin [23, 40]. Further, data obtained in animal models and humans suggest that OPN plays a role in the pathogenesis of multiple sclerosis, the most common demyelinating disease [15, 17, 31, 55], and has been suggested to enhance myelin formation in vitro [55]. The finding that OPN expression in preterm infant brains is associated only with WMI may further suggest an involvement of OPN in the process of white matter damage and/or repair. Therefore, further exploring the role of OPN in CNS development, white matter injury in preterm infants, and neurodevelopmental diseases will be beneficial for increasing our knowledge of these processes to develop novel therapeutic strategies.

OPN is also induced in other cell types in the brain where it leads to subsequent microglia activation. For example, in a mouse model of ischemic stroke, regulatory T cell-derived OPN acts through integrin receptors on microglia to enhance microglial activity and promote oligodendrogenesis [56]. OPN is involved in inducing the reactivity of astrocytes and is suggested to be involved in recovery and repair [29, 49]. In OPN^–/–^ mice and in macrophage-depleted mice, reactive astrocytes failed to properly extend processes toward the center of the infarction in a model of photothrombotic stroke [22]. It is likely that OPN is expressed by other cell types in the injured preterm brain that were not examined in the present study, and the cellular source might be influenced by the cause or origin of injury as well as by severity or time course of injury.

In conclusion, we observed OPN expression in microglia, astrocytes, and oligodendrocytes in human preterm brains, and elevated levels of OPN in microglia and astrocytes were associated with WMI but not with hemorrhage in the germinal matrix. Our findings further suggest that OPN may take part in the inflammatory process after WMI in the preterm brain. OPN is one of the most highly regulated proteins in multiple pathological conditions, including perinatal brain injury, but its expression and cellular source in human preterm brains, and its contribution to brain injury in preterm infants, are largely unknown. This study thus facilitates our understanding of OPN’s role under both physiological and pathological conditions in the human brain, and this may lead to greater elucidation of disease mechanisms and potentially better treatment strategies.

## Data Availability

The datasets used and/or analysed during the current study available from the corresponding author on reasonable request.

## Abbreviations

OPN: Osteopontin
WMI: White matter injury
PVWMI: Periventricular white matter injury
GMH: Germinal matrix hemorrhage
GMH-IVH: Germinal matrix-intraventricular hemorrhage
CNS: Central nervous system
H&E: Hematoxylin and eosin
PBS: Phosphate-buffered saline
ABC: Avidin–biotin complex
DAB: 3,3’-Diamino-benzidine
ROI: Region of interest
Iba1: Ionized calcium-binding adapter molecule 1
GFAP: Glial fibrillary acidic protein
